# The pathogenesis of multifocal osteonecrosis

**DOI:** 10.1038/srep29576

**Published:** 2016-07-11

**Authors:** Wei Sun, Zhencai Shi, Fuqiang Gao, Bailiang Wang, Zirong Li

**Affiliations:** 1 Centre for Osteonecrosis and Joint-preserving & Reconstruction, Orthopaedic Department, China-Japan Friendship Hospital, Beijing, 100029, China

## Abstract

Our objective was to study the incidence, etiology, and diagnosis of multifocal osteonecrosis (MFON) and its treatment options to facilitate an earlier diagnosis and to optimize treatment. A radiological investigation was performed in osteonecrosis patients with a high risk of MFON for a more accurate diagnosis between January 2010 and June 2015. For patients with osteonecrosis of both the hip and knee joints or for patients with a history of corticosteroid use or alcohol abuse who had osteonecrosis of one or more joints in the shoulder, ankle, wrist or elbow, magnetic resonance imaging (MRI) was also performed on other joints, regardless of whether these joints were symptomatic. Furthermore, we performed a radiological screening of 102 patients who had a negative diagnosis of MFON but were at a high risk; among them, another 31 MFON cases were successfully identified (30.4%). Thus, the incidence of MFON during the study period increased from 3.1% to 5.2%. Patients diagnosed with osteonecrosis and who are at a high risk of MFON should have their other joints radiologically examined when necessary. This will reduce missed diagnosis of MFON and facilitate an earlier diagnosis and treatment to achieve an optimal outcome.

Osteonecrosis is a disabling disorder that frequently occurs in the younger population aged from 20 to 50 years. This disease is compounded when it concurrently involves several other joints. Non-traumatic osteonecrosis most commonly involves the femoral head, and it may also involve other sites, such as the knee, shoulder, wrist and ankle joints and the long bone shaft. Multifocal osteonecrosis (MFON) is defined as a disease involving three or more separate anatomic sites concurrently or consecutively. For example, osteonecrosis of the hip, knee and shoulder or of the shoulder, knee and ankle are both MFON^1^. Osteonecrosis of bilateral femoral heads or knees is not considered MFON because only two separate anatomic sites are involved. MFON is a rare clinical condition that is reported in approximately 3% of osteonecrosis patients^2^. The most commonly reported risk factor for MFON is the use of a corticosteroid. Other risk factors include systemic lupus erythematosus (SLE), a human immunodeficiency virus infection, coagulation abnormalities (antiprothrombin III deficiency, protein S deficiency, factor V Leiden gene mutation and increased activity of the plasminogen activator), renal failure, inflammatory bowel diseases, multiple sclerosis, Sjogren’s syndrome, sickle cell disease, leukemia and lymphoma^2^,^3^,^4^,^5^,^6^,^7^,^8^,^9^,^10^,^11^,^12^,^13^,^14^,^15^,^16^. However, case reports of MFON have been limited, and there are a large number of MFON patients without any symptoms and who are thus difficult to diagnose. Therefore, the exact incidence of MFON among patients with various diseases remains unclear.

Severe acute respiratory syndrome (SARS) emerged in China during the first quarter of 2003. By the close of this outbreak in July 2003, many patients with SARS had received varying doses of steroid therapy, which were administered over various periods. In our general survey and diagnosis of osteonecrosis in post-SARS medical workers in Beijing from July 2003 to January 2004, 176 of the 551 medical workers were identified as having osteonecrosis, among whom 37 had MFON (21%)^3^. By contrast, between January 2005 and December 2009, only 17 of the 657 osteonecrosis patients under our care were diagnosed with MFON (2.6%). Hence, we suspected that there might be a missed diagnosis of MFON. From January 2010, we incorporated radiological examinations of the joints of patients at a high risk of osteonecrosis to facilitate an earlier diagnosis of MFON. In this study, we aimed to analyze the characteristics of MFON and its treatment methods by studying both post-SARS patients with MFON and MFON patients admitted to our center.

## Results

Thirty-seven of the 176 post-SARS osteonecrosis patients had MFON (21%). Between January 2005 and December 2009, only 17 of the 657 osteonecrosis patients under our care were diagnosed with MFON (2.6%). Between January 2010 and June 2015, only 48 of the 1507 osteonecrosis patients under our care were diagnosed with MFON (3.1%) upon admission. After the comprehensive evaluation of the joints of 102 patients who had a negative diagnosis of MFON but who were at a high risk, another 31 cases of MFON were successfully identified (30.4%). Consequently, the incidence of MFON during the study period increased to 5.2%. The MFON patients admitted to our center had a mean number of 5.7 osteonecrotic lesions, similar to the mean number of lesions (5.8) found in post-SARS MFON patients.

**Figure 1:**
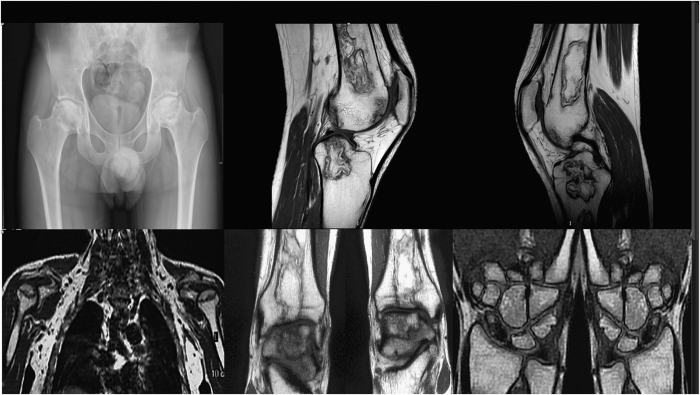
Radiological images of a male patient (age = 22 years). The patient had a history of corticosteroid therapy (equivalent dose of methylprednisolone = 7000 mg) for bone marrow transplantation due to acute lymphoblastic leukemia. He had pain in his hips but no symptoms in his other joints, and he was initially diagnosed with stage IIIc osteonecrosis of the bilateral femoral heads. The MRI examination of his other joints revealed osteonecrosis of the bilateral knees, shoulders, ankles and wrists.

The associated factors, diseases, and comorbidities are listed in Table 1. Of the 48 patients diagnosed with MFON on admission between 2010 and 2015, 31 were male and 17 were female. Forty-seven of the 48 MFON patients had a history of corticosteroid use, and the remaining one patient had a history of alcohol use. Of the patients with a history of corticosteroid use, 18 had SLE, nine had chronic nephropathy, five had hematological diseases (four had acute lymphoblastic leukemia and one had non-Hodgkin’s lymphoma), five had an organ transplantation (four had a renal transplantation and one had a cardiac transplantation), three had Sjogren’s syndrome, two had dermatomyositis, two had multiple sclerosis and three received steroid therapy for trauma emergency. Of the 31 newly diagnosed MFON patients, 19 were male and 12 were female. All of them had a history of corticosteroid use. Fifteen patients had SLE, eight had acute lymphoblastic leukemia, five had chronic nephropathy, and three had an organ transplantation (two had a renal transplantation and one had a hepatic transplantation). The MFON patients most commonly had osteonecrosis of the femoral head, followed by the knee, shoulder and ankle bones. The majority of patients had bilateral lesions (hips, knees and shoulders) (Fig. 1, Table 2). There was a significant difference in the number of MFON patients identified after the radiological screening based on risk factors compared with that before the screening (*P* < 0.05).

## Discussion

### Incidence of MFON

There have not been any journal articles in China concerning MFON, and such reports were also limited in other countries. Mont *et al*.^1^ studied 101 MFON cases diagnosed between 1980 and 1996 from 21 centers in the United States. According to the available data from 12 centers concerning their total number of patients with osteonecrosis, 81 patients had MFON of the 2484 patients (3.3%) diagnosed with osteonecrosis. In 2003, we screened post-SARS medical workers with corticosteroid use and found 37 patients had MFON of the 176 patients (21%) with a diagnosis of osteonecrosis. By contrast, between 2005 and 2009, we found only 17 patients with MFON of the 657 patients (2.6%) diagnosed with osteonecrosis who were admitted to our center. The incidence of MFON in post-SARS osteonecrosis patients was obviously higher than that in daily clinical practice. This is because the lesions found in joints, such as the knees, shoulders and ankles, in the early stages of MFON cases may not have any signs or symptoms. In our screening of post-SARS patients, all of them had a comprehensive MRI evaluation of their bilateral hips, knees, shoulders and wrists or ankles, which revealed the asymptomatic osteonecrosis. However, in daily clinical practice, we often focus on the osteonecrosis diagnosis of the femoral head without performing an MRI on the other joints. This implies that there are possible missed diagnoses of asymptomatic MFON patients. A recent study also showed a higher incidence of MFON than that typically reported in literature^4^.

### Etiology and distribution of osteonecrotic lesions

A few reports of MFON have suggested that a high dose of corticosteroids is the main risk factor for MFON. In France, Hernigou^4^ reported on 140 MFON cases diagnosed between 1985 and 1995, all of which were associated with corticosteroid use. In the cases reported by Mont *et al*.^1^, 91% had a history of corticosteroid use, and the rest had a coagulation disorder. Our study showed that 94 of the 96 (98%) MFON patients admitted to our center had a treatment history of high-dose corticosteroids, and the two remaining patients had a history of alcohol use. Moreover, the dosage and route of administration of the corticosteroids were obviously related to the incidence of MFON. A study by Hernigou^4^ demonstrated that the total dose and the daily dose of venous injection were closely related to the occurrence of MFON. This was also found in our study of post-SARS osteonecrosis patients caused by the use of corticosteroids^3^.

There have been a limited number of MFON case reports and a high occurrence of MFON in asymptomatic patients. Therefore, the exact incidence of MFON in patients with various diseases remains unclear. The incidence of MFON in 200 patients with sickle cell disease was reported to be 44% (87 of 200)^4^. MFON is recognized as a complication in the maintenance treatment of acute lymphocytic leukemia and non-Hodgkin’s disease, and its incidence in these diseases is also higher than that reported in the literature. Solarino *et al*.^9^ performed MRI screening in patients with acute lymphoblastic leukemia after chemotherapy and found that 82% of them had MFON^9^. In the MFON cases presented in this study, most were SLE, followed by hematological diseases, nephropathy, organ transplantation, dermatomyositis and multiple sclerosis. MFON was especially prevalent in leukemia patients; 17 of the 20 osteonecrosis patients with leukemia under our care were found to have MFON. Three of the four patients who received pulse steroid therapy for trauma emergency had a spinal cord injury, for which steroid therapy was considered appropriate. However, one patient received pulse steroid therapy for only an eye injury and was found to have osteonecrosis in eight joints, including the hips, knees, shoulders and ankles. Caution should be taken for such cases in the future.

MFON patients most commonly had osteonecrosis of the femoral head, followed by the knee, shoulder and ankle bones. Osteonecrosis of the shoulder, ankle and wrist never occurred aloneand was always accompanied by osteonecrosis of the hip and knee. Among the three populations of MFON patients presented in this study, 98–100% had femoral head involvement, 78–88% had knee involvement, and 29–67% had humeral head involvement. The average number of osteonecrotic lesions was 5.7 per patient.

### Diagnosis of MFON

To date, MRI is the most sensitive and specific tool to diagnose MFON. Initial studies found that low field MRI was as sensitive as a radionuclide scan in diagnosing osteonecrosis. Nevertheless, recent studies have demonstrated that high field (1.5–3 T) MRI has a higher sensitivity, specificity and accuracy in diagnosing osteonecrosis. Mont *et al*.^17^ compared the sensitivity of radionuclide scans with MRI, and found that MRI has a sensitivity of 100%. By contrast, the sensitivity of radionuclide scans is only 45%. Radionuclide scans have a higher sensitivity in detecting advanced stage osteonecrosis of the hip and knee than of the shoulder and ankle.

Although the diagnosis of osteonecrosis by MRI has its advantages, the screening for MFON with MRI is faced with a long scanning time and high cost. There have been debates regarding the optimal method to screen symptomatic and asymptomatic MFON^18^,^19^. Plain film X-ray is not sensitive enough to diagnose asymptomatic MFON, but there is still an advantage to carrying out an X-ray examination. Even if the X-ray result of a joint with pain is negative for osteonecrosis, it provides a preliminary examination of the local bone structure, which serves as a reference for further investigation by MRI. Other methods include a short time inversion recovery (STIR) MRI test followed by MRI or a radionuclide scan of specific joints. Nevertheless, further studies are required to determine the optimal method for early detection of osteonecrosis. Moreover, as the majority (70–100%) of osteonecrosis cases reported in the literature are bilateral, patients diagnosed with osteonecrosis of a joint on one side should be radiologically examined on the other side.

Missed diagnoses of MFON have frequently occurred in clinical practice. To reduce this, we recommend the following measures. 1) Patients with associated diseases and long-term use of high-dose corticosteroids should have their hips and knees evaluated by MRI within six to twelve months after the medication. 2) Patients diagnosed with osteonecrosis of the hip and knee should have their bilateral shoulders evaluated by MRI. 3) Patients with corticosteroid use or alcohol abuse and who are diagnosed with osteonecrosis of one or more joints in the shoulder, ankle, wrist and elbow should have their hips and knees evaluated by MRI.

### Treatment of MFON

MFON patients usually start with silent lesions. As the disorder gradually progresses, patients will experience joint pain. At first, the pain is mild and patients may only experience increased pain during movement or weight bearing on the affected bone or joint. As significant damage develops in the joints, patients will experience severe pain. The period of time between the first symptoms and the loss of joint function varies for each patient and ranges from several months to several years. In the late stages of MFON, patients often have symptoms at rest and reduced joint activity, resulting in severe joint dysfunction.

The treatment of MFON remains controversial. The general principle is to estimate the prognosis of each lesion. In addition, predicting the presence of collapse of the joint surfaces is the basis for determining the order of treatment. The progress of osteonecrosis is often different for each joint. Osteonecrosis of the femoral head progresses the fastest and attention should be paid to the early appearance of symptoms. This is followed by the progression of osteonecrosis to the knees and shoulders. Patients who have osteonecrosis of multiple weight-bearing joints and a large necrotic lesion area may develop joint destruction, and early intervention should be used. The progression of osteonecrosis to the elbow and wrist is relatively slow; however, we still lack experience regarding the appropriate treatment method for this area. Bone infarction often leads to calcification and self-repair. Hence, osteonecrotic elbows and wrists should mainly be managed by monitoring, and over treatment should be avoided if there are no clinical symptoms. In addition, an appropriate treatment strategy should be selected according to the stage of osteonecrosis in each joint involved^20^. For symptomatic patients at an early stage (i.e., ARCO Stages I, II or China Stages I–III), joint-preservation treatment options are commonly used, such as extracorporeal shock wave, core decompression, and vascularized and nonvascularized bone grafting. When >2 mm of the articular surface has collapsed or the necrotic lesion area is >50%, joint-preservation treatments may not provide good efficacy. In the advanced stages of the disease (i.e., ARCO Stages IIIc and IV, or China Stages V–VI), arthroplasty is required when joint preservation is not possible.

There are some limitations of this study. This study is limited by virtue of the retrospective analysis at only one center. And there was no randomized and blinded control group with conservative treatment in this study.

The incidence of MFON was high when clinical risk factors were present, such has high-dose steroid use, alcohol abuse, SLE, chronic nephropathy and leukemia. For a highly suspected case of MFON, a radiological screening of multiple joints is necessary, and MRI is still the gold standard for diagnosing MFON. Such screening can help to effectively reduce missed diagnoses. The goals of the treatment should be to take measures to delay the progress of the disease, to preserve the joint function and to avoid joint surface collapse and destruction by diagnosing the disease early. In addition, an appropriate treatment strategy should be selected according to the stage of MFON. Conservative treatment and joint-preservation treatment options should be adopted during the early stages, while arthroplasty should be performed during the advanced stages.

## Methods

This study was performed in accordance with the principles expressed in the Declaration of Helsinki and approved by the Ethics Committee of the China-Japan Friendship Hospital. Informed consent was obtained from all patients.

A standardized MRI was taken of patients, including their bilateral joints. The apparatus was a GE Sigma Profile/Gold, and T1 weighted, coronal STIR, and transverse T1WI sequences were used. The parameters of the scan included a layer thickness of 5 mm and a distance between layers of 5 mm. The T1WI were obtained with a TR from 416~440 ms and a TE of 15 ms, and the STIR images were obtained with a TR of 1512 ms and a TE of 80 ms. If an abnormal signal was found, then a T2 weighted scan was added, and for some of the patients, we also performed fat suppression and water-fat separation sequences. We only used T1 and T2 sequences for the other joints of the patients. Osteonecrosis was defined as either a subchondral or an intramedullary area demarcated by a distinct marginal rim with low signal intensity that encompassed the medullary fat on the MRI images. In addition to the MRI, all patients were subjected to an X-ray that included the affected joints.

Our general survey on the bones and joints of post-SARS medical workers who used corticosteroids revealed that the corticosteroid dose was approximately 2000 mg in unifocal osteonecrosis patients. By contrast, the dose was >5000 mg in all MFON patients except for one patient who received 2000 mg, with the highest dose at 31,000 mg. The MFON patients also had prolonged corticosteroid treatment (≥30 days)^3^. Of the 17 MFON patients diagnosed between January 2005 and December 2009, one had a 30-year history of alcohol abuse (average daily alcohol consumption of 500 g). The remaining 16 patients had a history of intensive steroid use for the following diseases: SLE (7 patients), acute lymphoblastic leukemia (4 patients), chronic nephropathy (3 patients), anaphylactoid purpura (1 patient) and pulse steroid therapy for traumatic shock (1 patient). Therefore, we summarized the high risk factors for MFON to include: a medical history of SLE, chronic nephropathy, hematological diseases or coagulation abnormalities (especially leukemia); total corticosteroid (methylprednisolone) dose >5000 mg, especially for patients with a history of intravenous pulse therapy; and a total corticosteroid administration time of >30 days.

Between January 2010 and June 2015, a total of 1507 osteonecrosis patients were admitted to our center, and only 48 of them were diagnosed with MFON (3.1%) upon admission. A radiological investigation was performed in patients with a negative diagnosis of MFON but who had a high risk of MFON and complaints of pain in other joints. For patients found to have osteonecrosis of both the hip and knee, MRI was performed on their bilateral shoulders and ankles, and also their bilateral wrists and elbows when necessary, regardless of whether other joints were symptomatic. For patients with a history of corticosteroid use or alcohol abuse and who were found to have osteonecrosis of one or more joints in the shoulder, ankle, wrist and elbow, MRI was performed on their hips and knees and their other joints when necessary.

The results were analyzed by a chi-square test the using SPSS 17 software. A *P* value < 0.05 was considered statistically significant.

## Additional Information

**How to cite this article**: Sun, W. *et al*. The pathogenesis of multifocal osteonecrosis. *Sci. Rep*. **6**, 29576; doi: 10.1038/srep29576 (2016).

## Figures and Tables

**Table 1:** The etiology of MFON.

	2010–2015 MFON diagnosed upon admission + after screening (48 + 31*)	2005–2009 (17)	Total (96)
SLE	18 + 15	7	40
Hematological diseases	5 + 8	4	17
Nephropathy	9 + 5	3	17
Organ transplantation	5 + 3	0	8
Sjogren’s syndrome	3 + 0	0	3
Dermatomyositis	2 + 0	0	2
Anaphylactoid purpura		1	1
Trauma	3 + 0	1	4
Multiple sclerosis	2 + 0		2
Alcohol use	1 + 0	1	2

*P < 0.05 from a chi-square test.

**Table 2:** Distribution of osteonecrotic lesions in MFON patients.

Osteonecrosis joint involvement	2010–2015 (79)*	2005–2009 (17)*	Post-SARS patients (37)*
Hip	155 (99%)	33 (100%)	72 (98%)
Knee	129 (82%)	30 (88%)	58 (78%)
Shoulder	106 (67%)	10 (29%)	33 (44%)
Ankle	54 (34%)	0	22 (9%)
Wrist	2 (2.5%)	0	7 (9%)
Patella	0	0	3 (4.5%)
Long bone shaft	4 (4%)	0	18 (45%)

*P < 0.05 for the comparison of lesion distribution among the three groups.
